# Comparison of up-front cash cards and checks as incentives for participation in a clinician survey: a study within a trial

**DOI:** 10.1186/s12874-020-01086-9

**Published:** 2020-08-17

**Authors:** Lydia E. Pace, Yeonsoo S. Lee, Nadine Tung, Jada G. Hamilton, Camila Gabriel, Sahitya C. Raja, Colby Jenkins, Anthony Braswell, Susan M. Domchek, Heather Symecko, Kelsey Spielman, Beth Y. Karlan, Jenny Lester, Daniella Kamara, Jeffrey Levin, Kelly Morgan, Kenneth Offit, Judy Garber, Nancy L. Keating

**Affiliations:** 1 Brigham and Women’s Hospital, 75 Francis St, Boston, MA 02115 USA; 2 Harvard Medical School, 25 Shattuck St, Boston, MA 02115 USA; 3 Beth Israel Deaconess Medical Center, 330 Brookline Ave, Boston, MA 02215 USA; 4 Memorial Sloan Kettering Cancer Center, 1275 York Ave, New York, NY 10065 USA; 5 Dana-Farber Cancer Institute, 450 Brookline Ave, Boston, MA 02215 USA; 6 Rush Medical College at Rush University, 600 S Paulina St Suite 202, Chicago, IL 60612 USA; 7 David Geffen School of Medicine at the University of California, 10833 Le Conte Ave, Los Angeles, CA 90095 USA; 8 Basser Center for BRCA, University of Pennsylvania, West Tower, Centre Square, 1500 Market St, Philadelphia, PA 19102 USA

**Keywords:** Clinician survey, Survey incentives, Response rate, Cash cards

## Abstract

**Background:**

Evidence is needed regarding effective incentive strategies to increase clinician survey response rates. Cash cards are increasingly used as survey incentives; they are appealing because of their convenience and because in some cases their value can be reclaimed by investigators if not used. However, their effectiveness in clinician surveys is not known. In this study within the BRCA Founder OutReach (BFOR) study, a clinical trial of population-based *BRCA1/2* mutation screening, we compared the use of upfront cash cards requiring email activation versus checks as clinician survey incentives.

**Methods:**

Participants receiving BRCA1/2 testing in the BFOR study could elect to receive their results from their primary care provider (PCP, named by the patient) or from a geneticist associated with the study. In order to understand PCPs’ knowledge, attitudes, experiences and willingness to disclose results we mailed paper surveys to the first 501 primary care providers (PCPs) in New York, Boston, Los Angeles and Philadelphia who were nominated by study participants to disclose their *BRCA1/2* mutation results obtained through the study. We used alternating assignment stratified by city to assign the first 303 clinicians to receive a $50 up-front incentive as a cash card (*N* = 155) or check (*N* = 148). The cash card required PCPs to send an activation email in order to be used. We compared response rates by incentive type, adjusting for PCP characteristics and study site.

**Results:**

In unadjusted analyses, PCPs who received checks were more likely to respond to the survey than those who received cash cards (54.1% versus 41.9%, *p* = 0.046); this remained true when we adjusted for provider characteristics (OR for checks 1.61, 95% CI 1.01, 2.59). No other clinician characteristics had a statistically significant association with response rates in adjusted analyses. When we included an interaction term for incentive type and city, the favorable impact of checks on response rates was evident only in Los Angeles and Philadelphia.

**Conclusions:**

An up-front cash card incentive requiring email activation may be less effective in eliciting clinician responses than up-front checks. However, the benefit of checks for clinician response rates may depend on clinicians’ geographic location.

**Trial registration:**

ClinicalTrials.gov (NCT03351803), November 24, 2017.

## Background

Surveying health care providers is an important means of obtaining information about medical practices and clinician knowledge and attitudes. However, clinician survey response rates in the United States have decreased gradually over time [1–3]. A 2013 meta-analysis described an approximately 20% decline in response rates over the preceding two decades [2]. The decline in response rates is thought to reflect increasing demands on clinicians’ time that limit participation in research activities [4]. Since low response rates can compromise study findings’ internal and external validity [5] and increase research costs, strategies to maximize clinician survey response rates are sorely needed.

The timing, type, and amount of monetary incentives provided to survey recipients are known to influence response rates [6]. A randomized study demonstrated higher clinician survey response rates with $50 versus $20 check incentives [6]. Timing of the incentive also impacts the likelihood of response, with up-front unconditional cash incentives yielding superior response rates compared with conditional cash incentives paid only after providers respond to the survey [7, 8] or lottery-based incentives [9]. Although cash cards and gift cards are increasingly used in survey research, little is known about their impact on clinician survey response rates. Cash cards have several potential advantages over cash or checks. Cash cards are increasingly used in day-to-day life, as people seek alternatives to cash or paper checks. In contrast to cash and similar to checks, some cash cards can be reclaimed by investigators if they are not used, although such cash cards require that unique cards or codes be assigned in advance to a specific survey recipient (i.e. registered) [10]. Because checks and registered cash cards can be tracked more easily than cash, they may be preferable to cash or non-registered cash cards for institutional accounting. Registered cash cards have the additional benefits of being logistically more feasible and efficient than checks, which must be generated individually for each clinician surveyed. Use of registered cash cards (hereafter called “cash cards”) for incentives have yielded adequate response rates in some studies [11, 12]. However, the impact of cash card incentives compared with other types of financial incentives on clinician survey response rates is not known.

We conducted a study comparing up-front, unconditional cash card survey incentives to check survey incentives to assess their impact on primary care provider (PCP) survey response rates. The BRCA Founder OutReach (BFOR) study is a clinical trial being conducted in New York, Boston, Philadelphia and Los Angeles examining the implementation of a digital platform and no-cost *BRCA1/2* founder mutation testing for individuals of Ashkenazi Jewish descent [13]. Study participants elect to receive *BRCA1/2* results from their PCP or a study-affiliated specialist. We surveyed PCPs elected by their patients to disclose results to determine PCPs’ knowledge, attitudes and experience with *BRCA1/2* testing and their willingness to disclose their patient’s results. In this substudy of survey incentives, we assigned PCPs to receive an up-front cash card or an up-front check incentive.

## Methods

### Survey

We surveyed the first 125 PCPs from each city who were elected by a BFOR participant to share his or her *BRCA1/2* results. Using a combination of questions derived from other surveys [14–16] and questions developed specifically for the BFOR project (Supplement), the survey gathered general demographic and practice information, assessment of *BRCA1/2* mutation knowledge, PCPs’ opinions on incorporating genetic testing into their existing practices, and willingness to disclose the results of their patients’ testing obtained through the BFOR study. We mailed paper surveys, although we also provided PCPs the option to participate via the Internet. Each initial survey mailing included a personalized cover letter, an up-front, unconditional $50 incentive, a four-page survey designed to be completed in less than 10 min, and a pre-paid return envelope. Surveys were prelabeled with each PCP’s assigned study identification number to allow study staff to identify which PCPs had already responded; no other identifiers were included in PCPs’ responses. First and second reminders were sent via mail roughly three and 6 weeks, respectively, after the initial mailing. These reminders contained personalized letters, a second copy of the survey, and a pre-paid return envelope.

### Incentive assignment and study sample

**Fig. 1:**
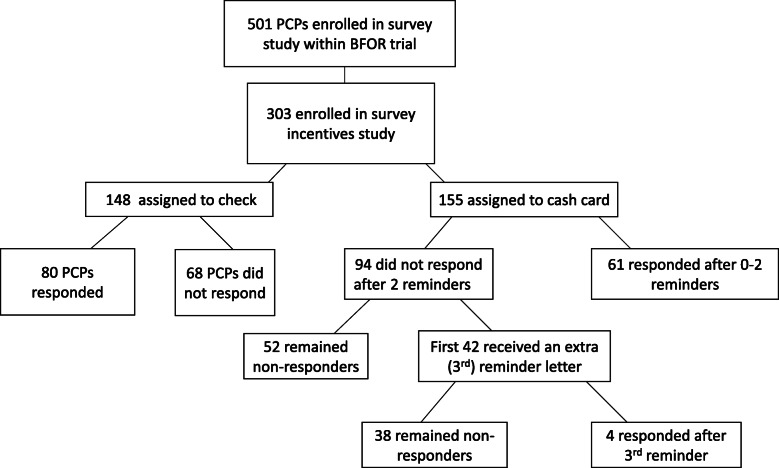
Enrollment of PCPs in the study and incentive assignment

Whenever a patient requested that their PCP disclose their results, that PCP immediately became eligible for the survey study and was assigned by research staff to receive $50 cash card or $50 check incentive. (Fig. 1) Assignment was performed as follow: as PCPs were nominated by their patients, they were assigned a study ID based on their region. Within each region, we used an alternating 1:1 allocation strategy to assign newly enrolled PCPs to receive a cash card or check. The cash cards were reloadable debit cards that required activation by the study managers before use. PCPs received instructions accompanying the card informing them the if they wished to activate the cash card they had to email a study manager with their card number and request card activation. In June 2018, in an effort to increase response rates, we additionally decided to send out third reminders to PCPs. The third reminder included a personalized letter, third copy of the survey, pre-paid return envelope, and a second incentive equivalent to the first. In June 2018, in an effort to increase response rates, we additionally mailed third reminders to PCPs. The third reminder included a personalized letter, third copy of the survey, pre-paid return envelope, and a second incentive equivalent to the first. We planned to send third reminders to all PCPs who had not yet responded to the survey, but third reminders were subsequently discontinued due to study staffing limitations. The third reminders were sent to the first 42 nonresponding PCPs who had been assigned to receive cash cards (providing them with a second cash card); third reminders were also planned for nonresponding PCPs who had been assigned to receive checks. However, issuing checks took more time than issuing cash cards, and the third reminder initiative was terminated before any third-reminder letters with checks were actually mailed. Our target sample size for the PCP survey study was 500 (125 per region), based on a sample size calculation derived from anticipated differences in factors influencing PCPs’ willingness to disclose their patients’ *BRCA1/2* results and a response rate of 50% derived from rates in other provider surveys [6, 17, 18]. After June 2018, due to overall response rates below 50% and early findings demonstrating that checks yielded higher response rates, the randomized study of incentives was stopped and all further survey mailings to newly enrolled PCPs included checks. This analysis includes the 303 PCPs enrolled in the randomized portion of the study between December 2017 and June 2018. Among these, 155 were assigned to receive their incentive in the form of a cash card and 148 were assigned to receive a check.

### Outcome and PCP characteristics

Our pre-specified primary outcome was response to the survey; staff determining this outcome were not blinded to the exposure. Our primary independent variable was receipt of a cash card versus check. Covariates were clinician city, specialty and sex; these were the demographic and practice data that were available for both responding and non-responding PCPs.

### Analysis

We used univariate Chi-square tests to examine response rates according to whether PCPs received checks or cash cards and according to PCP sex, city and specialty. Because more female PCPs were assigned to checks than males, we used multivariate logistic regression to adjust analyses for demographic characteristics. We also conducted stratified analyses of the impact of checks versus cash cards according to demographic characteristics, and we noted that the impact of checks versus cash cards appeared to vary by city. To explore this further, we incorporated interaction terms into our model. Finally, we conducted sensitivity analyses in which we reclassified as non-responding the 4 PCPs who had responded to the survey only after receiving a third survey reminder letter, since only a subset of PCPs received this third reminder. Statistical analyses were performed using SAS version 9.3 (Copyright 2002–2012, Cary, North Carolina).

This study adhered to the TREND checklist for reporting of nonrandomized studies (https://www.cdc.gov/trendstatement/). The BFOR study protocol is registered on clinicaltrials.gov (NCT03351803). The protocol for the survey incentives study described here was developed a priori subsequent to the overall BFOR study protocol and submitted to the institutional review board prior to participant enrollment.

## Results

Characteristics of surveyed PCPs are included in Table 1. Characteristics of PCPs who received checks versus cash cards did not differ by city, provider type, or specialty (*p* = 0.33, *p* = 0.23, and 0.09, respectively); however, a higher proportion of PCPs receiving checks were female (61.5% of check recipients versus 51.0% of cash card recipients; *p* = 0.03) (Table 1).

**Table 1:** Characteristics of primary care providers who received checks versus cash card incentives

	Received check (*n* = 148) N (%)	Received cash card (*n* = 155) N (%)	*p*-value*
**City**			0.33
Boston	47 (31.8%)	49 (31.6%)	
New York	44 (29.7%)	43 (27.7%)	
Los Angeles	33 (22.3%)	35 (22.6%)	
Philadelphia	24 (16.2%)	28 (18.1%)	
**Sex**			**0.03**
Male	57 (38.5%)	76 (49.0%)	
Female	91 (61.5%)	79 (51.0%)	
**Provider type**			0.23
Physician	145 (98.0%)	148 (95.5%)	
Advanced practice provider^a^	3 (2.0%)	7 (4.5%)	
**Specialty**			0.09
Internal Medicine (*n* = 207)	97 (65.5%)	110 (71.0%)	
Obstetrics & Gynecology (*n* = 48)	31 (21.0%)	17 (11.0%)	
Family Medicine (*n* = 44)	19 (12.8%)	25 (16.1%)	
Other (*n* = 4)^b^	1 (0.7%)	3 (1.9%)	

*Pearson’s chi-square tests

^a^includes 9 nurse practitioners and one nurse midwife

^b^Includes 3 surgeons and 1 radiation oncologist who were identified by patients as their primary care provider

Overall, 145 PCPs (47.9%) responded to the survey. Factors associated with survey response in unadjusted and adjusted analyses are shown in Table 2. In unadjusted analyses, survey response rates were higher among check recipients than cash card recipients (54.1% versus 41.9%, *p* = 0.046) and among female providers compared to males (53.5% versus 40.6%, *p* = 0.04). Advanced practice providers were more likely to respond than physicians (80.0% versus 46.8%, *p* = 0.04), although there were only 10 advanced practice providers surveyed. Response rates varied somewhat by city, with the highest response rates among providers from Boston (55.2%) and Philadelphia (48.1%), but differences across cities were not significant. Of the 42 PCPs who received third reminder letters with cash cards re-sent, only 4 (9.5%) responded to the survey. After adjustment for city, sex, provider type and specialty, PCPs receiving checks were more likely to respond to surveys than those receiving cash cards (OR 1.61 (95% CI 1.01, 2.59), *p* = 0.047; Table 2). No other provider characteristics were significantly associated with likelihood of response in the adjusted analyses. In the sensitivity analysis excluding the survey responses from the 4 PCPs who responded only after receiving third reminders, in multivariable logistic regression the association between receipt of a check and odds of survey response was stronger than in the main analysis (OR 1.82 (95% CI 1.13, 2.92)), *p* = 0.01) and advance practice providers had a greater adjusted odds of responding, though the confidence interval for the odds ratio was wide (OR 5.69 (95% CI 1.11, 29.11), *p* = 0.04).

**Table 2:** Unadjusted and adjusted response rates based on provider characteristics

Characteristic	*N*	*N* (%) responded	Unadjusted *p*-value*	Adjusted OR (95% CI)**	Adjusted *p*-value**
***Incentive***			**0.046**		
Cash card	155	65 (41.9%)		*Ref*	
Check	148	80 (54.1%)		1.61 (1.01, 2.59)	**0.047**
***Sex***			**0.04**		
Male	133	54 (40.6%)		*Ref*	
Female	170	91 (53.5%)		1.37 (0.84, 2.25)	0.20
***City***			0.33		
Boston	96	53 (55.2%)		*Ref*	
New York	87	37 (42.5%)		0.62 (0.34, 1.15)	0.13
Los Angeles	68	30 (44.1%)		0.64 (0.33, 1.24)	0.19
Philadelphia	52	25 (48.1%)		0.73 (0.36, 1.46)	0.37
**Provider type**			**0.04**		
Physician	293	137 (46.8)		*Ref*	
Advanced practice provider	10	9 (80.0)		4.80 (0.94, 24.39)	0.06
***Specialty***			0.65		
Internal Medicine	207	96 (46.4)		1.24 (0.62, 2.50)	0.54
Obstetrics & Gynecology	48	27 (56.3)		1.67 (0.69, 4.05)	0.26
Family Medicine	44	20 (45.5)		*Ref*	
Other	4	2 (50.0)		1.55 (0.19, 12.58)	0.68

*Chi-square tests

**multivariable logistic regression

Table 3 shows response rates among PCPs receiving checks versus cash cards stratified by city, and the results of the multivariable logistic regression model that included interaction terms for city and incentive type. The impact of incentive type on response rate varied notably by city (*p* = 0.02 using the Wald Chi Square test). In Boston and New York, the relationship between incentive type and survey response was not statistically significant. In Los Angeles and Philadelphia, checks were associated with statistically higher likelihood of survey response. In Los Angeles, 63.6% of those receiving checks responded to the survey versus 25.7% of those receiving cash cards (OR 4.73, 95% CI 1.64, 13.50), and in Philadelphia, 62.5% of those receiving checks responded versus 35.7% of those receiving cash cards (OR 3.61 (95% CI 1.11, 11.72). A sensitivity analysis removing the survey responses from the 4 PCPs who responded to the third reminders yielded similar results.

**Table 3:** Impact of providers’ city on the association between incentive type and likelihood of survey response

City	Survey responses among those receiving checks	Survey responses among those receiving cash cards	Adjusted OR for survey response with check versus cash card incentive (95% CI)^a^
Boston	53.2%	57.1%	0.74 (0.33, 1.69)
New York	43.2%	41.9%	1.07 (0.44, 2.58)
Los Angeles	63.6%	25.7%	**4.73 (1.65, 13.50)**
Philadelphia	62.5%	35.7%	**3.61 (1.11, 11.72)**

^a^Using logistic regression with interaction terms for city and incentive type; ORs are adjusted for provider sex, type and specialty. Measure of effect modification by city: *p* = 0.02 using Wald Chi-square test

## Discussion

In an era of declining clinician survey response rates, understanding the most successful and cost-effective strategies to optimize response rates is important for maximizing studies' validity and feasibility. Evidence about the effectiveness of cash cards for clinician surveys is very limited. In this randomized study, the overall PCP response rate was less than our goal of at least 50%, underscoring the persistent challenge of eliciting provider responses. However, among PCPs receiving checks, the overall response rate was 54.1%, compared to 41.9% among those receiving cash cards. The benefit of check incentives persisted when we adjusted for provider characteristics, suggesting that an upfront cash card incentive requiring email activation may be less effective in eliciting provider responses than up-front checks. However, the benefit of checks appears to be regionally specific: checks were associated with increased response rates in Los Angeles and Philadelphia, but not in Boston and New York. Another notable finding from our study was that advance practice providers such as nurse practitioners were more likely to respond to the survey, though the number of advance practice providers in our study was small and these findings need to be confirmed by additional research.

There are several potential explanations for our finding of a benefit of check incentives. PCPs may be more familiar with checks and feel that they are more straightforward to deposit and thus use. The need to email a study manager to activate the cash card may have also limited enthusiasm for this type of incentive. The regional differences may suggest that behaviors regarding cash cards and familiarity with them varies geographically. However, it is also possible that factors not related to incentive type contributed to our findings about differences between cities. For example, overall survey response rates were the highest in Boston, likely at least partly because the principal investigators for the PCP survey component of BFOR (who signed the survey cover letter) were Boston-based investigators. This difference may have attenuated some of the differential impact of checks versus cash cards in Boston, although it seems unlikely to fully explain the regional differences seen. Our findings suggest that investigators conducting local or regional surveys should consider local context when they choose survey incentives. For national studies, checks (or cash) may be a safer option to maximize survey responses.

Strengths of this study include its prospective enrollment of PCPs from 4 different cities. It also has some limitations. First, we used an alternating assignment strategy rather than randomization to assign providers to check versus cash card as providers were nominated by their patients to disclose results. We are not aware of any ways that this would have biased our findings in this unblinded study, however, and we used multivariable logistic regression to balance known confounders. Second, we had relatively limited covariates for non-responding PCPs, which limited the comparisons of responding and non-responding providers. Third, 42 PCPs, all in the cash card arm, received an intensified survey reminder approach, with a third reminder mailing enclosing a second incentive. Our sensitivity analyses demonstrated that this biased our findings somewhat towards the null. Fourth, we did not enroll as many PCPs as planned into this study of incentives because of our decision to use checks only for all PCPs after the 303rd PCP enrolled, in order to maximize the response rate and the corresponding robustness of our findings from the survey (which we will report separately). Lastly, our findings may not be generalizable to PCPs practicing outside major cities, or to other cities in the U.S. Nonetheless, we believe that these findings provide valuable information for researchers who are considering what types of incentives to use for provider surveys.

## Conclusion

Monetary incentives in the form of up-front checks may increase clinician survey response rates more than up-front cash card incentives. However, the differential impact of these incentives appears to be region-specific. Further research is needed to explore these differences. In addition, further research on cash card incentives for clinician surveys should explore whether not requiring email activation increases the effectiveness of the incentive, despite the possibility of added cost. Clinician surveys remain critical for understanding health care service delivery, and continued investigation is needed to identify the most effective and cost-effective strategies to optimize clinician survey response rates.

## Data Availability

The datasets used and analyzed during the current study are available from the corresponding author on reasonable request.

## Abbreviations

BRCA: BReast CAncer gene
PCP: Primary Care Provider
BFOR: BRCA Founder OutReach
